# The Role of Liuwei Dihuang Pills and Ginkgo Leaf Tablets in Treating Diabetic Complications

**DOI:** 10.1155/2016/7931314

**Published:** 2016-12-19

**Authors:** Yue Zhao, Jiangyi Yu, Jingshun Liu, Xiaofei An

**Affiliations:** Department of Endocrinology, Jiangsu Province Hospital of Traditional Chinese Medicine (The First Affiliated Hospital of Nanjing University of Chinese Medicine), Nanjing, China

## Abstract

*Objective.* To observe the clinical prophylactic and therapeutic efficacy of Liuwei Dihuang Pills and Ginkgo Leaf Tablets for type 2 diabetic vascular complications.* Methods.* It was a randomized, double-blind and placebo-controlled clinical trial. 140 outpatients with type 2 diabetes were recruited and randomly divided into the treatment group and control group. The two groups were given basic therapy (management of blood sugar, blood pressure, etc.). Additionally, the treatment group was given Liuwei Dihuang Pills and Ginkgo Leaf Tablets, while the control group was given Liuwei Dihuang Pills and Ginkgo Leaf Tablets placebos. All subjects were followed up for consecutive 36 months and observed monthly. The clinical data as urinary microalbumin to urinary creatinine ratio (Umalb/cr), carotid intima-media thickness (IMT), diabetic nephropathy (DN) and diabetic retinopathy (DR) prevalence, cardiovascular and cerebrovascular events, blood glucose, and blood pressure were collected and analyzed statistically.* Results.* After 36-month treatment, the Umalb/cr level and DN and DR prevalence in treatment group were all significantly lower than control group (*P* < 0.05). However, the IMT level and the incidence of cardiovascular and cerebrovascular events were not significantly different between the two groups (*P* > 0.05).* Conclusions.* Liuwei Dihuang Pills and Ginkgo Leaf Tablets are beneficial to diabetic microvascular complications, while the efficacy to diabetic macrovascular complications needs more observations.

## 1. Introduction

Diabetic vascular complications include two types: microvascular complications, such as diabetic nephropathy (DN), diabetic retinopathy (DR), and diabetic peripheral neuropathy (DPN), and macrovascular complications, including cerebrovascular disease, cardiovascular disease, and lower extremity artery disease. The relevant epidemiological surveys indicate that DN occurs in 20–40% of patients with diabetes and is the primary cause of end-stage renal disease [1]. DR is the leading cause of blindness among working-aged adults around the world [2]. DPN is one of the most frequently encountered complications of diabetes, affecting at least 50% of people with diabetes [3]. Furthermore, almost 50% of patients with type 2 diabetes will develop heart failure [4]. Foot ulcers and amputation, which are consequences of diabetic neuropathy and/or peripheral arterial disease (PAD), are common and represent major causes of morbidity and mortality in people with diabetes [1].

Clinicians mostly focus on the intervention and control of diabetes complications. “Compared with treatment, prevention matters much.” It is a consensus on clinic that the occurrence of complications could be effectively prevented with positive intervention in the early stage of diabetes [5]. How to choose the appropriate preventive interventions? Is there any other effective method in addition to lifestyle intervention, management of blood glucose, blood pressure, blood lipids, and other risk factors [6–8]? Many Western medical researchers made a lot of efforts on it; meanwhile traditional Chinese medicine experts also did much corresponding work [9–12]. What benefits could Chinese medicine intervention bring to the patients of diabetic vascular complications? Therefore, a randomized, double-blind and placebo-controlled study was performed and analyzed on the intervention of type 2 diabetes patients by traditional Chinese medicine to provide evidence for these questions.

## 2. Methods

### 2.1. Study Design

It was a randomized, double-blind, double-simulated and placebo-controlled clinical trial. The patients with type 2 diabetes were outpatients recruited from the Department of Endocrinology of Jiangsu Province Hospital of Traditional Chinese Medicine. The recruited patients all met the following criteria:

1. Type 2 diabetes mellitus between 50 and 75 years old; blood pressure < 160/100 mmHg; and body mass index (BMI) < 35 kg/m^2^.
2. No or only mild microvascular complications (e.g., urinary microalbumin to urinary creatinine ratio < 220 mg/g, nonproliferative diabetic retinopathy).
3. No diagnosed macrovascular complications (e.g., coronary heart disease, cerebrovascular accident, and lower limb vascular lesions).
4. No serious systemic diseases (e.g., cancer, severe inflammation, AIDS, and mental disease).
5. No bilateral cataract, no allergic constitution, and no pregnant or lactating women.
6. Completely understanding and signing informed consent (ethical number 2007 NL008).

These subjects were randomly divided into treatment group and control group with 70 subjects each. All the patients underwent a complete history collection (age, sex, diabetes duration, etc.), physical examination (height, weight, blood pressure, etc.), determination of blood biochemistry (blood glucose, cholesterol, etc.), and basic characteristics collection (therapeutic regimen, lifestyle, etc.) after recruitment. Patients of both groups were given basic treatment with reference to “Chinese type 2 diabetes prevention guideline (2007 edition)” including management of blood glucose and blood pressure, health education, diet instruction, and related indexes monitoring. Additionally, the treatment group was given Liuwei Dihuang Pills (oral, 8 pills/time, 3 times a day; batch number Z41022128; Wanxi Pharmaceutical Co., Ltd.) and Ginkgo Leaf Tablets (oral, 2 tablets/time, 3 times a day; batch number Z20027949; Yangtze River Pharmaceutical Co., Ltd.). Meanwhile, the control group was given placebo of Liuwei Dihuang Pills and Ginkgo Leaf Tablets (made by the same factory and there was no difference with real drugs in the morphological appearance). All subjects accepted an on-site visit on clinic once a month for testing related indexes, guiding treatment, distributing and taking back research drugs, and so forth. The researchers and subjects were both blind to group division which was finally revealed after consecutive 36-month observation. The related data before and after treatment were collected and analyzed statistically.

Main observation indexes and test instruments were as follows:

1. Urinary microalbumin to urinary creatinine ratio (Umalb/cr) was tested by the automatic biochemical analyzer (Olympus AU2700).
2. Diabetic retinopathy degree was evaluated by ophthalmoscope (Welch Allyn REF13010) and retina camera (KOWA Nonmyd7).
3. Carotid intima-media thickness (IMT) was tested by the colored ultrasonic machine (ESAOTE-MyLab 70 XVision).
4. The fasting blood glucose (FBG), postprandial blood glucose (PBG), glycosylated hemoglobin (HbA1c), total cholesterol (TC), triglyceride (TG), high density lipoprotein (HDL), and low density lipoprotein (LDL) were tested by the automatic biochemical analyzer (Olympus AU2700).
5. The incidence of cardiovascular and cerebrovascular events was judged and diagnosed by clinical doctors.

Research case termination criteria were as follows:

1. Severe adverse events, allergy, or other adverse reactions.
2. Serious deviation of trial plan (e.g., poor medication compliance) that researchers thought it difficult to evaluate drugs effect.
3. Subjects who did not want to continue in the process of trial and applied for withdrawing from the study.

### 2.2. Statistical Analysis

Values were expressed as means ± SD if they were in accordance with normal distribution; otherwise, the data was converted or modified with statistical method. Measurement data was analyzed by *t*-test; meanwhile data was counted by chi-square test or Fisher's exact test. It was two-sided test, and *P* < 0.05 was considered to be statistically significant. SAS 9.1 software (SAS Institute Inc., Cary, North Carolina) was used for all statistical analyses.

### 2.3. Ethics

The program was approved by the Ethics Committee of Jiangsu Province Hospital of Traditional Chinese Medicine (approval number 2007NL008). A written informed consent was obtained from every subject.

## 3. Results

In this study, 140 subjects were recruited and randomly divided into treatment group and control group. As shown in Tables 1 and 2, there were no significant differences of demographic characteristics (sex, age, height, weight, and waist circumference) and vascular complication risk factors (smoking, diet control, physical exercise, health education, glucose monitoring, duration, history, and family history) between the two groups (*P* > 0.05). Meanwhile, there were no significant differences of DN prevalence, DR prevalence, cardiovascular events, and cerebrovascular events between the two groups before the treatment as shown in Table 3  (*P* > 0.05).

25 cases were terminated during the 36-month treatment period including 4 cases automatic quit, 1 case poor compliance, 1 case drug reaction, and 5 cases adverse events (2 cases liver cirrhosis, 2 cases cerebral infarction, and 1 case coronary heart disease) in treatment group, while 7 cases were automatic quit, 0 cases poor compliance, 2 cases drug reaction, and 5 cases adverse events (1 case endometrial cancer, 1 case focal liver lesions, 1 case cerebral hemorrhage, 1 case cerebral infarction, and 1 case coronary heart disease) in control group. There were no significant differences in case termination between the two groups as shown in Table 4 (*P* > 0.05).

Before the treatment, there were no significant differences of blood glucose, blood pressure, and blood lipid between the two groups. The same results were also found after 36-month treatment as shown in Table 5 (*P* > 0.05)

Furthermore, there were no significant differences of UmAlb/cr and IMT between the two groups before the treatment and the same result was also shown in IMT after the treatment. However, the treatment group UmAlb/cr was significantly lower than control group after the 36-month treatment (27.5 ± 52.1 mg/g versus 39.3 ± 52.3 mg/g, *P* < 0.05) as shown in Table 6.

The DR prevalence of treatment group was significantly lower than control group after the 36-month treatment (8.5% versus 25.0%, *P* < 0.05). The DN prevalence of treatment group was also significantly lower than control group after the 36-month treatment (16.9% versus 30.4%, *P* < 0.05). However, there were no significant differences of cerebrovascular and cardiovascular events between the two groups after treatment  (*P* > 0.05) (Table 7).

After 36-month treatment, the basic treatments in both groups including blood glucose and blood pressure managements were analyzed. There were no significant differences of hypoglycemic agents and hypotensive drugs between two groups as shown in Tables 8 and 9.

## 4. Discussion

It has been increasingly accepted that Chinese medicine plays an effective role in treating diabetes and its complications for a long time [13–15], which still need further stronger clinical evidences to support. With this aim, the 36-month, randomized, double-blind, placebo-controlled and prospective clinical study was planned, operated, and analyzed scrupulously in our research.

In this research, the drugs applied for diabetes treatment were two kinds of Chinese patent medicine including Liuwei Dihuang Pills and Ginkgo Leaf Tablets. Liuwei Dihuang Pills were comprised of Rehmanniae radix praeparata, Corni fructus, Dioscoreae rhizoma, Alismatis rhizoma, Moutan cortex, and Poria (the proportion was 8 : 4 : 4 : 3 : 3 : 3) and every 8 pills were equivalent to 3 g of original medicinal materials. Ginkgo Leaf Tablets consisted of ginkgo leaf extract containing 9.6 mg of flavonoids glycol glycosides and 2.4 mg of terpenoid lactones in each piece.

Diabetes shares similar pathogenesis with ancient disease “Xiaoke” in traditional Chinese medicine. The main syndrome of “Xiaoke” is Yin deficiency and blood stasis, and the corresponding therapeutic method is to nourish Yin and promote blood circulation [9], which was also the reason that Liuwei Dihuang Pills and Ginkgo Leaf Tablets were chosen among many classical Chinese medicines in this research [16, 17]. Besides, it was also attributed to the following. (1) Modern pharmacological study confirmed that the relevant components of Liuwei Dihuang Pills (Rehmanniae radix praeparata, Corni fructus, Dioscoreae rhizoma, Alismatis rhizoma, Moutan cortex, and Poria) played the effective roles in anti-inflammation, antioxidation, immune regulation, and cardiovascular protection [18–22]. Meanwhile, the ginkgo leaf extract also played a part in the inhibition of platelet aggregation, antioxidation, dilation of blood vessel, and improving the abnormal blood rheology [23–26]. These pharmacological characteristics might be beneficial to the prevention of diabetic vascular complications [27, 28]. (2) The two drugs belonged to Chinese patent medicines with the advantages of stable composition, convenient storage and taking, and good adherence which ensured higher scientificity and repeatability to the study results.

In this study, there were no significant differences of demographic characteristics (sex, age, height, weight, and waist circumference), complication risk factors (smoking, diet control, physical exercise, health education, glucose monitoring, duration, history, and family history), DN prevalence, DR prevalence, and cardiovascular and cerebrovascular events between the two groups before treatment. The two groups were of good comparability because all the above-mentioned conditions were equal before treatment; meanwhile, there were no significant differences of case termination between the two groups during the trial period. There was no situation against implementation plan with good quality control in the process of research in treatment group. Therefore, the results were scientific and reliable.

After 36-month treatment, the UmAlb/cr level and DN and DR prevalence in treatment group were significantly lower than those in control group. Therefore, it was suggested that Liuwei Dihuang Pills and Ginkgo Leaf Tablets could effectively retard the development of type 2 diabetic nephropathy and diabetic retinopathy. In contrast with the UKPDS results that intensive glycemia control could reduce 21% risk of DR and 33% risk of DN as observed in 12-year period [29, 30], the results in this study indicated that Chinese medicine intervention can significantly reduce 66% risk of DR prevalence (8.5% versus 25.0%) and 44% risk of DN prevalence (16.9% versus 30.4%) in 3-year period, which suggested that integrative Chinese and Western medicine treatment was more beneficial and valuable in preventing diabetic microvascular complications than pure western medicine.

However, the similar benefit was not found in diabetic macrovascular complications prevention. The study results showed that there were no significant differences of IMT and cerebrovascular and cardiovascular events between the two groups after treatment. Was it real that Chinese medicine intervention was not effective to diabetic macrovascular complications? There were still some doubts on it in this study: both cerebral apoplexy and coronary heart disease incidences were relatively low in the two groups (1.6%~3.4%). These diabetes patients might not be in obvious occurrence period of macrovascular complications, and it was not the best observation point in reality in three years. The evidence to support the above view might be more convincing if the observation period was extended much longer.

In summary, the study was a three-year period, large sample, randomized, double-blind and placebo-controlled clinical research. The research was strictly executed with the reference of implementation plan to get real and reliable results, which provided important clinical evidence to prove the viewpoint that Chinese medicine had better effect in diabetic vascular complications prevention and treatment.

## 5. Conclusions

Chinese medicine treatment (Liuwei Dihuang Pills and Ginkgo Leaf Tablets) is beneficial to diabetic microvascular complications including diabetic retinopathy and diabetic nephropathy. However, the impact of Liuwei Dihuang Pills and Ginkgo Leaf Tablets to diabetic macrovascular complications needs further observations.

## Figures and Tables

**Table 1:** Comparison of demographic characteristics between two groups.

Group	Case number	Sex	Age (years)	Height (cm)	Weight (kg)	Wc (cm)	BMI (kg/m^2^)
		Male/female					
T	70	39/31	62.2 ± 6.4	164.6 ± 8.9	65.3 ± 10.2	84.8 ± 8.1	24.1 ± 3.0
C	70	31/39	60.7 ± 6.5	163.0 ± 7.8	63.8 ± 7.4	83.3 ± 8.9	24.0 ± 2.6

T: treatment, C: control, Wc: waist circumference, BMI: body mass index.

**Table 2:** Comparison of vascular complication risk factors between two groups.

Group	Case number	Smoking	Diet control	Physical exercise	Health education	Glucose monitoring	DM duration (years)	DM family history	CHD family history	Hypertension history	Hyperlipidemia history
		Never/ever/now	Never/common/good	Never/occasionally/usually	Yes/no	Never/once a month/more than once a month		Yes/no	Yes/no	Yes/no	Yes/no
T	70	53/2/15	5/37/28	10/12/48	54/16	10/23/37	6 ± 4	28/42	10/60	34/36	37/33
C	70	62/1/7	6/34/30	6/23/41	60/10	6/21/43	6 ± 5	27/43	13/57	34/36	38/32

T: treatment, C: control, DM: diabetes mellitus, CHD: coronary heart disease.

**Table 3:** Comparison of prevalence of DR, DN, cerebrovascular, and cardiovascular events between two groups before treatment.

Group	Case number	DR		DN		CA		CHD	
		No	Yes	No	Yes	No	Yes	No	Yes
T	70	65	5 (7.1%)	58	12 (17.1%)	70	0 (0.0%)	70	0 (0.0%)
C	70	68	2 (2.9%)	56	14 (20.0%)	70	0 (0.0%)	70	0 (0.0%)

T: treatment, C: control, DR: diabetic retinopathy, DN: diabetic nephropathy, CA: cerebral apoplexy, CHD: coronary heart disease.

**Table 4:** Comparison of case termination between two groups after treatment.

Group	Case number	Terminated case	Cause of termination			
			Automatic quit	Poor compliance	Drug reaction	Adverse events
T	59	11 (15.7%)	4	1	1	5
C	56	14 (20.0%)	7	0	2	5

T: treatment, C: control.

**Table 5:** Comparison of blood glucose, blood pressure, and blood lipid between two groups before and after treatment.

Group	Time	Case number	FBG (mmol/L)	PBG (mmol/L)	HbA1c (%)	SBP (mmHg)	DBP (mmHg)	TC (mmol/L)	TG (mmol/L)	HDL-C (mmol/L)	LDL-C (mmol/L)
T	Before	70	7.1 ± 2.2	10.7 ± 4.3	6.5 ± 1.3	129 ± 13	76 ± 9	4.8 ± 0.8	1.5 ± 1.0	1.3 ± 0.3	2.8 ± 0.7
	After	59	6.8 ± 1.4	8.8 ± 1.6	6.4 ± 1.1	122 ± 11	76 ± 6.5	5.1 ± 1.0	1.6 ± 1.2	1.3 ± 0.3	2.9 ± 0.7
C	Before	70	7.1 ± 1.8	9.8 ± 3.4	6.6 ± 1.4	130 ± 14	77 ± 9	4.8 ± 0.9	1.5 ± 0.9	1.3 ± 0.3	2.9 ± 0.6
	After	56	6.8 ± 1.3	9.2 ± 1.7	6.3 ± 0.8	122 ± 12	77 ± 6	4.9 ± 0.9	1.3 ± 0.6	1.2 ± 0.3	2.8 ± 0.7

T: treatment, C: control, SBP: systolic blood pressure, DBP: diastolic blood pressure.

**Table 6:** Comparison of UmAlb/cr and IMT between two groups before and after treatment.

Group	Time	Case number	UmAlb/cr (mg/g)	IMT (mm)
T	Before	70	24.0 ± 12.5	0.66 ± 0.13
	After	59	27.5 ± 52.1	0.68 ± 0.16
C	Before	70	25.6 ± 17.7	0.67 ± 0.13
	After	56	39.3 ± 52.3^*∗*^	0.68 ± 0.14

T: treatment, C: control.

^*∗*^ *P* < 0.05, T versus C.

**Table 7:** Comparison of prevalence of DR, DN, cerebrovascular, and cardiovascular events between two groups after treatment.

Group	Case number	DR		DN		CA		CHD	
		No	Yes	No	Yes	No	Yes	No	Yes
T	59	54	5 (8.5%)	49	10 (16.9%)	59	2 (3.2%)	59	1 (1.6%)
C	56	42	14 (25.0%)^*∗*^	39	17 (30.4%)^*∗*^	56	2 (3.4%)	56	1 (1.7%)

T: treatment, C: control, DR: diabetic retinopathy, DN: diabetic nephropathy, CA: cerebral apoplexy, CHD: coronary heart disease.

^*∗*^ *P* < 0.05, T versus C.

**Table 8:** Comparison of hypoglycemic agents between two groups.

Group	Case number	Hypoglycemic agents						
		Metformin	Sulfonylureas	Glinides	Acarbose	Thiazolidinedione	Insulin	Insulin-analog
T	59	41 (69.5%)	39 (66.1%)	3 (5.1%)	11 (18.6%)	7 (11.9%)	7 (11.9%)	11 (18.6%)
C	56	35 (62.5%)	40 (71.4%)	2 (3.6%)	14 (25.0%)	8 (14.3%)	6 (10.7%)	8 (14.3%)

T: treatment, C: control.

**Table 9:** Comparison of hypotensive drugs between two groups.

Group	Case number	Hypotensive drugs					
		*β*RB	CCB	ACEI	ARB	Diuretic	Others
T	59	2 (3.4%)	19 (32.2%)	3 (5.1%)	2 (3.4%)	2 (3.4%)	3 (5.1%)
C	56	3 (5.3%)	20 (35.7%)	2 (3.6%)	2 (3.6%)	3 (5.3%)	3 (5.3%)

T: treatment, C: control, *β*RB: *β* receptor blockers, CCB: calcium channel blockers, ACEI: angiotensin converting enzyme inhibitors, ARB: angiotensin II receptor blockers.

## Additional Points

This was a randomized, double-blind and placebo-controlled clinical trial about Chinese medicine applied in diabetic complications. It was concluded that Chinese medicine treatment was beneficial to diabetic microvascular complications. The efficacy of Chinese medicine in diabetic macrovascular complications treatment needs further observations.
